# Editorial: New advances in the field of nerve regeneration

**DOI:** 10.3389/fneur.2025.1541159

**Published:** 2025-02-05

**Authors:** Ouwen Qiu, Wenchuan Zhang

**Affiliations:** Department of Neurosurgery, School of Medicine, Shanghai Ninth People's Hospital, Shanghai Jiao Tong University, Shanghai, China

**Keywords:** nerve regeneration, peripheral nerve injury, diagnostic innovation, molecular mechanism, cellular mechanism

Nerve regeneration remains a significant challenge in neurology and regenerative medicine, given the complexities of restoring both structural and functional recovery in injured nerve tissues. Recent advancements, spanning novel diagnostic tools, therapeutic strategies, and cellular-level insights, highlight a collective effort to address these challenges. This editorial synthesizes key findings from groundbreaking research published in the Research Topic, *New Advances in the Field of Nerve Regeneration, Frontiers in Neurology* (2023–2024), emphasizing their impact on advancing the understanding and treatment of peripheral nerve disorders.

## Diagnostic innovations: from imaging to screening

Accurate diagnosis is foundational to effective treatment. Chen, Zou, et al. highlight the utility of B-mode ultrasound imaging in diagnosing carpal tunnel syndrome, with its ability to visualize structural changes such as nerve swelling and flattening, providing hand surgeons with a non-invasive auxiliary tool to enhance accuracy. Complementing this, Dong et al. explore advanced imaging modalities for peripheral nerve injury, emphasizing their potential to refine injury characterization. Meanwhile, Zhou et al. provide a large-scale analysis of spinal muscular atrophy carrier screening, emphasizing the significance of early identification in preventing genetic disorders.

## Therapies for peripheral nerve injuries

Therapeutic interventions have seen notable progress, particularly in nerve regeneration. Liu Z. et al. investigate repetitive transcranial magnetic stimulation (rTMS) for peripheral facial paralysis, demonstrating efficacy differences based on stimulation sites. Xu et al. review advancements in autologous peripheral nerve transplantation, underscoring strategies to optimize recovery, and also discusses emerging technologies, such as bioengineered grafts and nerve conduits, that enhance functional outcomes. Furthermore, Zou, Dong, et al. discuss techniques and graft materials for repairing peripheral nerve defects, and highlight the importance of combining advanced biomaterials with precise surgical approaches to improve functional recovery in patients with severe nerve injuries, offering insights into overcoming surgical challenges.

## Cellular and molecular mechanisms in nerve regeneration

Understanding cellular interactions and molecular pathways is crucial for advancing nerve repair. Jiang et al. delve into the interplay between Schwann cells and the extracellular matrix, shedding light on their pivotal roles in regeneration. Zhang et al. explore kinase signaling in peripheral nerve repair, revealing therapeutic targets that may revolutionize regenerative strategies. Aisaiti et al. extend this perspective with a bibliometric analysis on mesenchymal stem cells, identifying trends and gaps in stem cell-based therapies for nerve repair.

## Emerging concerns: nitrous oxide and cosmetic surgery

Unique challenges in peripheral neuropathy are also receiving attention. Zou, Yi, et al. review nitrous oxide-induced neuropathy, elucidating mechanisms and advocating for heightened awareness in clinical practice. Similarly, Chen, Li, et al. investigate nerve injuries post-cosmetic surgery, offering recommendations to minimize risks and improve outcomes.

## Mapping the research landscape

Visualization studies provide a macroscopic view of research trends. Wang et al. analyze sciatic nerve injury treatment, identifying key research frontiers and facilitating targeted innovation. This aligns with efforts by Liu G. et al., who propose a modified approach for lumbar interbody fusion, enhancing surgical outcomes for spinal conditions linked to nerve injuries.

## Conclusion

The collective efforts highlighted in these studies underscore a vibrant era of progress in peripheral nerve research. From novel diagnostic tools and therapeutic approaches to deepening insights into molecular mechanisms, the field is advancing toward improved patient outcomes. As this body of work continues to grow, interdisciplinary collaboration will remain crucial to overcoming remaining challenges and translating these findings into clinical practice.

